# Assessment of rehabilitation effects in children with mild intellectual disability

**DOI:** 10.1038/s41598-023-42280-1

**Published:** 2023-09-20

**Authors:** Andżelina Wolan-Nieroda, Anna Wojnarska, Grzegorz Mańko, Aleksandra Kiper, Agnieszka Guzik, Andrzej Maciejczak

**Affiliations:** 1 Department of Physiotherapy, College of Medical Sciences, Institute of Health Sciences, University of Rzeszów, Rzeszów, Poland; 2 Students Scientific Club for Physiotherapy in Adults’ and Children’s Neurology, Medical College of the University of Rzeszów, Rzeszów, Poland; 3 Department of Biomechanics and Kinesiology, Institute of Physiotherapy, Jagiellonian University Collegium Medicum, Krakow, Poland; 4 ORNR “Krzeszowice”, Rehabilitation Center, Krzeszowice, Poland; 5 Doctoral School of the University of Rzeszów, Rzeszów, Poland; 6 Institute of Medical Sciences, College of Medical Sciences, University of Rzeszów, Rzeszów, Poland

**Keywords:** Neurodevelopmental disorders, Paediatrics

## Abstract

Research on effectiveness of rehabilitation programmes continues to investigate impact of therapeutic interventions on various motor parameters in children with intellectual disability (ID). This study compared the effectiveness of rehabilitation, reflected by physical fitness, static balance, and dynamic balance measurements, in children with mild ID. A total of 70 children with mild ID were enrolled for the study and were divided into two equal groups based on their body mass index (BMI) percentile, reflecting obesity or normal weight. Physical fitness was assessed using the Eurofit Special Test, whereas balance was evaluated with single-leg stance and timed up and go tests. The examinations were performed twice: At the beginning and at the end of a six-month therapy programme. Improvements were shown in the muscle strength of the upper limbs (*p* < 0.001) and lower limbs (*p* = 0.001), flexibility (*p* = 0.005), and static balance (*p* < 0.001) for the entire cohort. The effects of rehabilitation did not differ significantly between the children with obesity and those with a normal weight. These results may be important from the viewpoint of clinical practice and preventive measures, as they present evidence showing that rehabilitation is equally effective in both obese and normal weight children with mild ID. Therefore, these findings may be of assistance to those designing therapeutic programmes in special education centres.

## Introduction

Intellectual disability (ID) is defined as a spectrum of disorders and abnormalities in the intellectual, physical, motor, emotional, and social domains^1,2^. The term "pure form" of intellectual disability refers to the case where the person affected is characterised solely by intellectual impairment, without accompanying additional developmental or medical disorders. In this situation, intellectual deficit is the only major factor impacting the person's functional status, whereas his/her physical capacities, health and other aspects do not present abnormalities. The current study focused on children with mild ID, classified as "pure form ID", in order to better understand the impact of rehabilitation on this specific group of children. Children affected by this condition experience a variety of challenges related to learning, communication, social skills and independent functioning^1–3^. Furthermore, existing reports suggest that children with ID are most likely to have problems with balance, coordination, endurance, flexibility, and muscle strength^4–15^. For many of them, appropriate rehabilitation may be an effective tool to enable improvement in the quality of life and functioning. Rehabilitation designed for children with ID mainly focuses on improving their motor skills, as well as self-care and social skills, in order to enable their social engagement and to foster their independence^6–9^.

Researchers focusing on effectiveness of rehabilitation programs continue to investigate impact of therapeutic interventions on various motor parameters in children with ID. Muscle strength, balance and dexterity play a key role in daily activities and self-care. Likewise, muscular endurance, speed, and flexibility are key elements impacting the ability to perform a variety of motor activities^4,5,9–19^. Furthermore, it may be important to understand in what way these motor parameters are affected by body mass index (BMI). Excessive body weight, particularly obesity, can affect muscle strength, flexibility and endurance, which can impact the overall effectiveness of therapeutic interventions^6–10^. A review of the related publications focusing on these issues showed that there are few studies investigating the gains from rehabilitation programmes intended for children with "pure form of ID^4,6,9^. Moreover, the relationship between BMI and effects of rehabilitation administered to children with ID has rarely been investigated. The only studies available assess the effect of BMI on the motor capacities exhibited by these children^6–9,12^.

Therefore, we envisage that our study will contribute evidence of key importance for understanding the comprehensive impact of rehabilitation on different aspects of functioning in children with ID, and may provide information which will help design therapies matching the specific needs of each child. With the results obtained, therapists and specialists will be able to create personalised therapy programmes that comprehensively support the motor development and quality of life in children with ID.

The present study aimed to assess the effects of rehabilitation programme on the static balance and dynamic balance in children with mild ID, and on their physical fitness reflected by such motor parameters as upper and lower limb muscle strength, speed, flexibility and muscular endurance. Additionally, the study was designed to investigate whether the effects of rehabilitation differ between children, depending on their BMI.

Research questions:

- 1. Does a six-month rehabilitation programme produce a change in the lower and upper limb muscle strength, speed, flexibility, and local muscular endurance in children with mild ID in both the obesity group (O Group) and normal weight group (NW Group)?
- 2. Does participation in a six-month rehabilitation programme affect static and dynamic balance in children with mild ID in both O and NW Groups?
- 3. Do the effects of rehabilitation differ between children with mild ID and obesity (O Group) and children with mild ID and normal weight (NW Group)?

## Methods

### Participants

This study was carried out in a special educational facility in the Podkarpackie Region in Poland. A total of 70 children with mild ID were enrolled for the study, and they were allocated to two equal-size groups relative to their BMI. O Group comprised 35 children with mild ID and obesity, including 17 boys and 18 girls, with a mean age of 12.9 ± 1.38 years. The age- and sex-matched NW Group consisted of 35 children with mild ID and normal weight. The characteristics of the two groups are shown in Table 1.

**Table 1:** Baseline characteristics of O and NW Groups.

	O Group (N = 35)	NW Group (N = 35)
Age (years), mean (SD)	12.9 (1.38)	12.2 (1.13)
Sex (female/male)	18/17	18/17
Height (cm), mean (SD)	149.3 (3.2)	152.5 (3.8)
Weight (kg), mean (SD)	66.1 (2.1)	41.2 (2.4)
BMI percentile, mean (SD)	99 (2.2)	58.2 (1.8)
Wechsler scale	68 (3.3)	67 (3.2)

*SD* standard deviation, *BMI* body mass index, *N* number of subjects.

All of the children recruited for the study met these inclusion criteria: (a) mild ID; (b) age between 11 and 15 years; (c) BMI percentile matching one of the two body mass categories: obesity > 95th percentile (O Group) or normal weight > 5th and < 85th percentile (NW Group); (d) attendance of a special educational facility; (e) no motor disability; (f) participation in a uniform rehabilitation programme; (g) a health status allowing for participation in the examinations.

The exclusion criteria were as follows: (a) moderate to severe ID, with cognitive deficits impairing their ability to understand and follow instructions; (b) age under 11 or over 15 years; (c) BMI classified as overweight (> 85 and < 95 percentile) or underweight (< 5 percentile); (d) lack of parents' and/or legal guardians' informed consent for their children’s participation; (e) coexisting conditions: autism, Down syndrome, cerebral palsy, muscular dystrophy, or neurological disorders such as epilepsy or brain damage; (f) comorbidities such as rheumatic, orthopaedic, oncological, or cardiac diseases.

### Procedures

The children were qualified for the study based on the diagnosis of mild intellectual disability issued by the Psychological and Pedagogical Counselling Centre, where they had been assessed by a qualified psychologist experienced in working with individuals with ID. During that assessment process, the children’s intellectual capacities were measured using Wechsler Test^20^. Wechsler Intelligence Scale is a highly recognised psychometric test commonly used to evaluate intelligence. It was designed by David Wechsler to measure various aspects of the cognitive function. There are a few versions of the tool, adequate for different age groups, e.g., Wechsler Intelligence Scale for Children (WISC) and Wechsler Adult Intelligence Scale (WAIS). They comprise subtests assessing a number of cognitive domains such as verbal capacities (e.g., vocabulary, comprehension) and non-verbal capacities (e.g., pattern recognition, spatial thinking)^20^.

The children’s BMI percentile values were determined using the calculator developed in the OLAF project^21^. Based on these values, the children were assigned to matching body mass categories, in line with the classification applicable to children and adolescents, as adopted by the Centers for Disease Control and Prevention (CDC)^22^. Children with obesity were assigned to O Group, and those with normal body weight were included in NW Group.

An assessment of physical fitness and balance in the children from both groups was performed twice: At the start of the rehabilitation programme (Exam 1) and at the end of the six-month therapy programme (Exam 2). The measurements were carried out in a gym with the necessary equipment, i.e., gymnastic bench, mattresses, medicine balls, two 150 cm-tall marker flags, chair, stopper, and measuring tape. During the tests, the children were wearing sports attire that did not restrict their movements. Before the testing, a 10 min all-round warm-up was carried out. The specific test trials were conducted according to instructions, in a defined order. Each trial was preceded by an introduction, explanation and demonstration.

The experimental protocol was approved by the Scientific Research Ethics Committee of the University of Warmia and Mazury in Olsztyn (approval no. 9/2018). All of the procedures were executed in full compliance with the principles set forth in the Declaration of Helsinki. Parents or legal guardians of all the children were informed about the purpose of the study and provided written consent for their inclusion in the study.

### Rehabilitation programme

The children in O and NW Groups participated in the therapy conducted with the same intensity and for the same duration of time. Rehabilitation sessions (45 min in duration), were held three times a week over a period of six months. The sessions in both groups followed the same schedule (Table 2). The therapy focused on improving functional strength, local muscular endurance and flexibility, static and dynamic balance, motor coordination and body schema, as well as spatial orientation. The programme consisted of introductory warm-up exercises, as well as aerobic exercise on static equipment (treadmill or bicycle), exercise strengthening postural muscles, balance and coordination exercises, breathing exercises, and exercises to develop body schema (getting to know one's own body by touching, looking at one's reflection in a mirror, showing and naming body parts, imitating the movements of another person) and spatial orientation. In addition to the therapy, the children in O and NW Groups participated in the regular activities and classes scheduled in the school, i.e., speech therapy and music, as well as visual and related arts.

**Table 2:** Description of the therapy sessions.

Type of exercise	Duration per one session
Introductory warm-up, aerobic exercise on electric treadmill	15 min
Balance and strength exercises improving muscular flexibility and endurance, with the use of 0.5 kg weights, elastic exercise bands, and balance discs	20 min
Relaxation and breathing exercises	10 min
Duration of therapy per week: Three times per week for 45 min each	

### Outcome measures

The following research tools were used in the study: The Eurofit Special Test^23,24^, the single-leg stance test with eyes open and closed^25^, and the timed up and go (TUG) test^26^.

The Eurofit Special Test is designed to assess the physical fitness of individuals with ID. It is a well-tested and reliable tool, commonly used to evaluate the overall fitness of children with ID^23,24^. It consists of six trials which assess: (1) dynamic balance—walking on gymnastic bench in the upright position (score expressed in points), children, assessed subjectively, could receive between 1 and 6 points with a higher score reflecting a better result; (2) lower limb muscle strength – standing long jump (in cm), measurements were taken to the nearest 1 cm, and a higher score reflected greater lower limb muscle strength; (3) upper limb muscle strength—a 2 kg medicine ball forward push with one hand (cm), measurements were taken to the nearest 1 cm, a higher score reflected greater upper limb muscle strength; (4) speed—a 25 m run from high start (in seconds), the time was measured in seconds to the nearest 0.1 s, a lower score meant that the designated distance was covered faster; (5) flexibility—seated forward bend (in points), measurements were taken to the nearest 1 cm, a higher score recorded from the '0' position reflected greater flexibility; (6) local muscular endurance—bent knee sit-ups (score ex-pressed in number) in 30 s, more repetitions reflected a better score^23,24^.

The single-leg stance test assesses static balance, measuring how long an individual can stand on one leg without support, with eyes open, and then with eyes closed. During the test, the subject stands barefoot on one leg while simultaneously bending the other knee backwards 90°, maintaining the thigh in a vertical position parallel to the standing leg. Separate measurements are taken for the right and left leg. The longer the balance is maintained, the better the result (recorded in seconds)^25^.

The timed up and go test is used to assess dynamic balance and independent mobility. It measures the time needed by an individual to get up from a chair, walk 3 m, complete a 180° turn, and return to the chair. This test is a popular balance assessment tool, and is therefore often used in clinical practice^26^.

### Sample size

A minimum sample size (Nmin) of 58 subjects was determined for the population studied using a sample size calculator (“PLUS module” from Statistica 13.3 software). Overall, O Group and NW Group included 70 children. Nmin for the present study was calculated using the following formula:

$$N\min = NP(\alpha 2 \cdot f\left( {1 - f} \right))$$

$$NP \cdot e2 + \alpha 2 \cdot f\left( {1 - f} \right)$$

where NP is total number of the population from which the sample is drawn; α represents the level of confidence for the results; f denotes the fraction size; and e stands for the anticipated maximum error.

### Data analysis

Statistical analyses were computed using Statistica 13.3 software from StatSoft. Parametric and non-parametric tests were applied. A given test was selected if its assumptions were met, for instance, the normality of distributions of the variables was assessed using the Shapiro–Wilk *W* test, and verified using Kolmogorov–Smirnov test. The changes in the results over time (before and after therapy) were assessed using Student’s *t* test for dependent variables, or alternatively with Wilcoxon’s signed-rank test. Differences in the effects of the therapy obtained in the two groups were assessed using Student's *t* test for independent variables or alternatively with the Mann–Whitney U-test. The therapy effect was defined as the value of the difference between the result obtained after the therapy relative to the result obtained before the therapy. The results of the parametric tests are presented as the descriptive statistics of the mean values and standard deviation, whereas the results of the non-parametric tests are presented as the descriptive statistics of median value, as well as of the first and third quartile. A value of p < 0.05 was assumed to reflect statistical significance.

## Results

### Research question 1: assessment of physical fitness in the whole cohort

Analysis of the scores achieved in the Eurofit Special Test by children with MID for the entire cohort, before and after the rehabilitation programme, showed statistically significant differences in the following measures: lower limb muscle strength in the standing long jump, upper limb muscle strength in the 2 kg medicine ball forward push with one hand, and flexibility in the seated forward bend. The scores in the trial assessing lower limb muscle strength after the therapy improved by 6.7 on average, compared to the measurement taken before the start of the rehabilitation programme, and this difference was statistically significant at *p* < 0.001. The improvement in the children’s upper limb muscle strength at the end of the therapy is shown by the scores, which, on average, increased by 5.9, and the difference between the variables was statistically significant at a level of *p* < 0.001. In the measure of flexibility, the improvement observed at the end of rehabilitation is reflected by a mean increase in the score by 1 cm compared to the results identified before the therapy, and the result was statistically significant at a level of *p* < 0.005. Detailed data are shown in Table 3. On the contrary, the assessments performed using the Eurofit Special Test showed no statistically significant changes in the scores identified before and after therapy for dynamic balance (*p* = 0.535), speed in the 25 m run test (*p* = 0.153), or local muscular endurance (*p* = 0.156) (Table 3).

**Table 3:** Differences between the scores in the Eurofit Special Test before and after the rehabilitation programme for the entire cohort.

	Rehabilitation	Mean ± SD/Median (Q1–Q3)	*p* (Student’s *t* test/Wilcoxon’s test)
Walking on a gymnastic bench in the upright position (points)	Before	5.0 (4.0–6.0)	0.535
	After	5.0 (4.0–6.0)	
	Effect	0.0 (–1.0 to 0.0)	
Standing long jump (cm)	Before	120.0 ± 38.04	< 0.001
	After	126.7 ± 38.79	
	Effect	6.7 ± 6.49	
2 kg medicine ball forward push with one hand (cm)	Before	273.5 (226.0–320.0)	0.001
	After	265.5 (235.0–343.0)	
	Effect	5.5 (3.0–9.0)	
25 m run from high start (s)	Before	5.4 (4.9–5.7)	0.153
	After	5.5 (4.9–6.0)	
	Effect	0.0 (0.0–0.1)	
Seated forward bend (points)	Before	27.0 (25.0–30.0)	0.005
	After	30.0 (25.0–30.0)	
	Effect	0.0 (0.0–2.0)	
Sit-ups (number of repetitions)	Before	10.0 (9.0–13.0)	0.156
	After	10.0 (10.0–13.0)	
	Effect	0.0 (0.0–1.0)	

*p*—significance level.

### Research question 2: assessment of static and dynamic balance in the entire cohort

Analysis of the results achieved by the entire cohort in the single-leg stance test before and after therapy showed significant changes in the scores for the trials with eyes open. On average, the scores improved by 5.2 s in the trials on the right leg, and by 5.7 s in the trials on the left leg. The identified differences were statistically significant at a level of *p* < 0.001. The results reflecting static balance were also improved in the trials with eyes closed. The mean score improved by 2.3 s for the right leg, and by 2.4 s for the left leg. These differences were statistically significant (*p* < 0.001). The assessment of dynamic balance with the timed up and go test, performed before and after the rehabilitation programme, showed no statistically significant differences between the variables (*p* = 0.345). Detailed data are shown in Table 4.

**Table 4:** Static balance (single-leg stance with eyes open and closed) and dynamic balance (timed up and go): differences between the scores before and after the rehabilitation programme for the entire cohort.

	Rehabilitation	Median (Q1–Q3)	*p* (Wilcoxon’s test)
Single-leg stance (s) (right leg, eyes open)	Before	14.7 (7.0–19.9)	< 0.001
	After	20.0 (19.6–20.0)	
	Effect	2.2 (0.1–10.6)	
Single-leg stance (s) (left leg, eyes open)	Before	13.6 (4.6–20.0)	< 0.001
	After	20.0 (19.6–20.1)	
	Effect	2.8 (0.1–10.8)	
Single-leg stance (s) (right leg, eyes closed)	Before	5.0 (2.6–6.1)	< 0.001
	After	7.0 (6.1–7.7)	
	Effect	2.0 (1.2–3.2)	
Single-leg stance (s) (left leg, eyes closed)	Before	4.1 (3.0–5.3)	< 0.001
	After	7.1 (5.2–8.0)	
	Effect	2.7 (1.1–3.5)	
Timed up and go (s)	Before	5.6 (5.2–6.2)	0.345
	After	5.8 (5.3–6.2)	
	Effect	0.0 (0.0–0.1)	

*p*—significance level.

### Research question 3: differences in the scores before/after and in the effects of rehabilitation between O and NW groups

The subsequent statistical analyses were intended to identify the differences in the scores before and after the rehabilitation programme, and in the effects of rehabilitation between O and NW groups, as reflected by the measurements of physical fitness (scores in the Eurofit Special Test specific trials), static balance (scores in single-leg stance test), and dynamic balance (scores in the timed up and go test).

First, a comparison of the groups was performed before the intervention and the only significant difference (*p* < 0.05) was identified in dynamic balance (timed up and go test); more specifically NW group was found with better dynamic balance before rehabilitation compared to O group. Conversely, the measurement of physical fitness (scores in the Eurofit Special Test specific trials) and static balance (scores in single-leg stance test) showed that before the intervention there were no statistically significant differences between the scores of O Group and NW Group (*p* > 0.05). Detailed data are shown in Table 5 and 6.

**Table 5:** Differences in the scores before and after the programme and in the effects of rehabilitation, reflected by the Eurofit Special Test physical fitness scores achieved by O and NW Groups.

	Rehabilitation effect	Mean ± SD/Median (Q1–Q3)	*p* (Student’s *t* test/Mann–Whitney *U*-test)
Dynamic balance	NW group-Before	5.0 (4.0 to 6.0)	0.886
	O group-Before	5.0 (3.0 to 6.0)	
	NW group-After	5.0 (4.0 to 5.0)	0.766
	O group-After	5.0 (4.0 to 5.0)	
	NW group-Effect	0.0 (–1.0 to 0.0)	0.718
	O group-Effect	0.0 (–1.0 to 0.0)	
Lower limb muscle strength	NW group-Before	118.2 ± 34.2	0.791
	O group-Before	121.8 ± 42.5	
	NW group-After	125.9 ± 36.1	0.903
	O group-After	127.5 ± 42.4	
	NW group-Effect	7.6 ± 5.07	0.405
	O group-Effect	5.8 ± 7.69	
Upper limb muscle strength	NW group-Before	251.0 (226.0–294.0)	0.163
	O group-Before	297.0 (230.0–388.0)	
	NW group-After	269.0 (229.0–290.0)	0.088
	O group-After	293.0 (249.0–399.0)	
	NW group-Effect	4.0 (2.0–7.0)	0.116
	O group-Effect	7.0 (4.0–11.0)	
Speed	NW group-Before	5.32 ± 0.74	0.567
	O group-Before	5.48 ± 0.87	
	NW group-After	5.37 ± 0.78	0.617
	O group-After	5.52 ± 0.90	
	NW group-Effect	0.0 ± 0.15	0.754
	O group-Effect	0.0 ± 0.10	
Flexibility	NW group-Before	27.0 (25.0–30.0)	0.647
	O group-Before	27.0 (25.0–30.0)	
	NW group-After	30.0 (25.0–30.0)	0.681
	O group-After	30.0 (26.0–30.0)	
	NW group-Effect	0.0 (0.0–2.0)	0.888
	O group-Effect	0.0 (0.0–2.0)	
Local muscular endurance	NW group-Before	10.0 (9.0–10.0)	0.130
	O group-Before	13.0 (9.0–14.0)	
	NW group-After	10.0 (10.0–11.0)	0.063
	O group-After	12.0 (10.0–14.0)	
	NW group-Effect	0.0 (0.0–1.0)	0.197
	O group-Effect	0.0 (0.0–0.0)	

*p* significance level.

**Table 6:** Relationship between the scores before and after the programme and in the effects of rehabilitation on static balance (single-leg stance test with eyes open or closed) and dynamic balance (timed up and go test) achieved by O and NW Groups.

	Rehabilitation effect	Median (Q1–Q3)	*p* (Mann–Whitney *U*-test)
Single-leg stance (s) (right leg, eyes open)	NW group-Before	10.0 (7.0–19.8)	0.344
	O group-Before	18.4 (8.1–20.0)	
	NW group-After	20.0 (19.7–20.0)	0.770
	O group-After	20.0 (19.6–20.0)	
	NW group-Effect	5.9 (0.1–11.9)	0.370
	O group-Effect	1.3 (0.1–5.6)	
Single-leg stance (s) (left leg, eyes open)	NW group-Before	9.0 (4.2–20.0)	0.318
	O group-Before	15.4 (10.2–20.0)	
	NW group-After	20.0 (19.9–20.1)	1.000
	O group-After	20.0 (19.5–20.1)	
	NW group-Effect	8.2 (0.0–12.2)	0.630
	O group-Effect	2.2 (0.1–5.9)	
Single-leg stance (s) (right leg, eyes closed)	NW group-Before	5.0 (2.1–5.2)	0.361
	O group-Before	5.5 (3.2–6.8)	
	NW group-After	7.0 (6.0–7.1)	0.174
	O group-After	7.3 (6.3–8.1)	
	NW group-Effect	1.9 (1.2–2.9)	0.310
	O group-Effect	2.8 (1.3–3.9)	
Single-leg stance (s) (left leg, eyes closed)	NW group-Before	3.8 (2.7–5.0)	0.249
	O group-Before	4.5 (3.3–6.3)	
	NW group-After	6.3 (5.2–7.2)	0.050
	O group-After	7.9 (6.2–8.2)	
	NW group-Effect	2.0 (1.1–3.9)	0.449
	O group-Effect	2.9 (1.9–3.5)	
Timed up and go (s)	NW group-Before	5.3 (5.0–6.1)	0.040
	O group-Before	6.1 (5.5–6.3)	
	NW group-After	5.3 (5.1–6.0)	0.031
	O group-After	6.0 (5.5–6.4)	
	NW group-Effect	0.0 (–0.0 to 0.0)	0.448
	O group-Effect	0.0 (–0.1 to 0.0)	

*p* significance level.

Similarly, the measurements carried out after the intervention showed significant differences (*p* < 0.05) only in dynamic balance (timed up and go test); the scores achieved by NW group reflected better dynamic balance after rehabilitation compared to O group. On the other hand, the scores in physical fitness (Eurofit Special Test specific trials) and static balance (single-leg stance test) showed no statistically significant differences between O Group and NW Group (*p* > 0.05) after the intervention. Detailed data are shown in Table 5 and 6.

Finally, the analyses focusing on the effects of rehabilitation reflected by the children’s physical fitness assessed using the specific trials of the Eurofit Special Test, showed no statistically significant differences between the effects achieved by O Group and NW Group (*p* > 0.05) Detailed data are shown in Table 5. Similarly, no statistically significant differences in the effects of rehabilitation (*p* > 0.05) were found between O Group and NW Group in the measures of static balance (single-leg stance test with eyes open/closed) or dynamic balance (timed up and go test)—Table 6.

## Discussion

The present study was designed to assess effectiveness of a rehabilitation programme in a group of children with mild ID. It assessed various aspects, such as physical performance and balance, to gain better understanding of the impact of rehabilitation in the case of children with mild ID. Additionally, the assessment aimed to determine whether or not rehabilitation effects differ relative to the children's BMI. A review of the related publications focusing on these issues showed that there are few studies investigating the gains from rehabilitation programmes intended for children with a “pure form” of ID^4,5,11–16,18,19^. The current study additionally adopted the approach which took into account children with mild ID and normal weight or obesity. The authors believe that this is the added value of this study, compared to earlier reports focusing on this subject matter^6–9^.

Another difference between our study and other research reports lies in the fact that we designed and used a comprehensive therapy programme focusing on multiple aspects. This programme was based on clinical experience of work with children with ID presenting with both obesity and normal weight. The programme comprised aerobic and anaerobic exercise, along with elements of strength and balance practice, as well as relaxation exercise. This is a novelty in this field of research, since the vast majority of the studies reported earlier applied strictly targeted interventions (e.g., trampoline exercise^14^, hippotherapy^15^, jumping rope^16^, strength training^17^, or rhythmic gymnastics^18^). In fact, the above observations provided the motivation for the present study, which we believe contributes evidence that is important for use in clinical practice and in designing therapy programmes for children with ID.

First, the current study investigated the possible changes in physical fitness of an entire cohort of children with mild ID participating in a six-month rehabilitation programme. The findings showed that the programme contributed to better muscle strength in the lower extremities and arms, as well as better flexibility. The study by Golubović et al. also assessed physical fitness in children with ID in comparison to normally developing peers. Functional arm strength was measured using a flexed-arm hang test, while lower limb strength was assessed using a long jump test. The latter measure was the same as that in the current study. Although the results within the groups were not statistically significant, follow-up trials assessing the long-term effects of exercise showed the most visible improvement in strength in the children with ID participating in a six-month programme^18^. Another study, carried out by Xu et al., assessed muscle strength in the lower and upper limbs using a standing long jump test and a dumbbell press test. In this case, children with ID participated in a four-month adapted rhythmic gymnastics programme, whereas the control group took part in conventional physical education classes. The researchers reported that the children in the study group were found with significantly improved muscle strength in both the upper and lower limbs, whereas the improvement in the control group, identified in the lower limb muscle strength, was less pronounced^5^. Kachouri et al. assessed lower limb strength using a dynamometer and found improvements in muscle strength in the group participating in the training^27^. Similarly, Giagazoglou et al. reported that a three-month exercise programme provided to children with ID produced an increase in lower limb muscle strength and improved flexibility, assessed with a sit and reach test^15^, as in the present study. The outcome measure of flexibility in children with ID was also positively affected by a three-month sports games programme, as reported by Pejčić et al.^28^, and by a three-month jumping rope training programme applied in the study by Chao-Chien et al.^16^.

In contrast, the present study identified no significant changes in two motor characteristics at the end of the rehabilitation programme, i.e., running speed and local muscular endurance. We assume that the lack of improvement in running speed could be explained by the fact that speed is a motor characteristic that can only be improved to a small degree, as it is highly dependent on a person’s innate disposition^28^. Furthermore, it has been reported that, in comparison to the general population, individuals with ID typically achieve a lower running speed or speed of limb movement^29^. In the present study, local muscular endurance was assessed with a sit-up test. The same approach was applied by Golubović et al., who, in fact, found an improvement in this measure in children with ID following an exercise programme, yet the differences between the baseline and final assessment were not statistically significant^11^. The results in our study could be linked to the elements of our rehabilitation programme. An improvement in muscular endurance was not a primary focus of the programme, which only contained elements of endurance exercise as an addition to balance, strength, and other exercise.

The current study also investigated the effect of the six-month rehabilitation programme on static and dynamic balance in the entire cohort of children with mild ID. Dynamic balance was assessed in two ways: during one of the Eurofit Special Test trials, which involved walking on a gymnastic bench in the upright position, and with the up and go test. No changes in this measure were identified by these two tools at the end of the programme. Static balance was assessed using the single-leg stance test with eyes open and closed, and the trials showed significant positive changes in the scores at the end of the therapy. Different findings were reported by Golubović et al., who assessed static balance using the Flamingo Balance Test in a study group participating in a six-month exercise programme and in two control groups comprising children with ID and healthy children, matched for age, sex, and skills, not included in the training programme. In this case, no statistically significant differences between the first and second measurement were observed within the specific groups^11^. A potential improvement in the dynamic balance of children with mild ID was assessed by Giagazoglou et al.^15^. Children in the study group participated in a three-month exercise programme, whereas the control group attended physical education classes twice a week, in line with the school schedule. In this case, the researchers reported a significant improvement in balance in the study group. The differences in the scores between the first and second measurements in the latter group were statistically significant^15^. On the contrary, Dehghani et al. investigated the effectiveness of balance training^30^. Static and dynamic balance were assessed using a subtest of the Bruininks–Oseretsky Test of Motor Proficiency. Children assigned to the study group took part in a 10-week balance training programme, whereas the control group continued to follow the regular school schedule. The results of this study revealed significant differences between the pre- and post-intervention values in the study group for both static and dynamic balance^31^. Results similar to ours were acquired by Kachouri et al., who investigated the effectiveness of a two-month strength and proprioceptive training programme in children with mild ID. The authors reported improvements in balance in the group of children participating in the therapy^27^. Importantly, the current study revealed considerable improvements, both in the childre’'s lower limb muscle strength and in static balance following the rehabilitation programme. We believe that this observation is consistent with the findings of another study that showed a correlation between balance and leg strength in children with mild ID^32^. In their study, Jeng et al. carried out a physical fitness follow-up in children with cerebral palsy undertaking a 12-week individualised exercise training regime. They showed that, compared to the control group, the follow-up group demonstrated better muscle strength, agility, and balance^32^. However, in our study, a positive change was only found in static balance, possibly linked to improved lower limb muscle strength, but no such change was identified in the measure of dynamic balance. It has been suggested by other researchers that static balance affects dynamic balance; however, our findings do not support this claim^29,30,33–38^. Although increased muscle strength beneficially impacts both types of balance, it is likely that an improvement in dynamic balance specifically also requires greater mobility^29,30,33,36^. Our rehabilitation programme included more exercises aimed at improving stability than mobility, which possibly explains this result. Furthermore, we know from clinical experience that it is very difficult for obese children with ID to perform dynamic balance exercises.

From the viewpoint of therapy, children with ID are a difficult group, as they tend to exhibit little interest in, or motivation for, exercise. Due to this, in future studies, it would be advisable to redesign the therapy programme by incorporating some incentivising and motivating elements, and by adding more exercises aimed at improving dynamic balance. For this purpose, various types of complex exercise programmes could be used. Mikołajczyk et al. proposed a programme comprising dual-task functional exercises performed on an unstable surface. These exercises increase the effectiveness of postural muscles, improve postural reflexes, and stimulate postural control in adolescents, contributing to improved dynamic balance^38^.

Finally, in line with the third detailed research question, this study compared the scores achieved by O Group and NW Group before and after the intervention in order to identify the effects of rehabilitation. The comparison of the groups before and after the intervention showed the only statistically significant differences in dynamic balance (timed up and go test); in both cases NW Group achieved better scores than O group. However no statistically significant differences in the effects of rehabilitation were found between O Group and NW Group, as reflected by the median for effects of rehabilitation amounting to zero in both groups. No statistically significant differences in the scores before and after the therapy and in the effects of rehabilitation were found between O and NW Groups in the measures of physical fitness (Eurofit Special Test specific trials) as well as static balance (single-leg stance test with eyes open/closed). It is likely that the differences in the dynamic balance, shown by the comparative assessment of the groups before the intervention, may be associated with the facts that body weight affects biomechanics of body movement as well as muscle strength^39^, and obesity-related limitations in joint mobility affect the ability to maintain balance^39,40^. Co-existing obesity leads to changes in the way a person moves and interacts with the ground, which has a negative effect on the ability to maintain dynamic balance^40,41^.

It is in fact possible to find research reports discussing the effects of various training programmes. However, in those cases the authors primarily focused on the factor of intellectual disability (e.g., its severity). As there are no reports on cohorts similar to ours (i.e., children with mild ID and with obesity or normal weight), we are unable to discuss our findings by reference to other similar works. The findings showed no significant differences between these two groups with regard to the effects of rehabilitation reflected by physical fitness or by static or dynamic balance. We suspect that this is because the children in O Group wanted to keep up with their normal-weight peers, and so participated in the rehabilitation programme with great commitment. However, we did not find any published research reports with related findings for discussion or to verify the results of our study. A review of the literature, however, showed that the relationship between BMI and physical fitness has been investigated. The effects of BMI on physical fitness, or more precisely on muscular endurance, muscle strength, flexibility, and aerobic capacity, were examined by Frey et al.^12^. The study showed that BMI affects muscle strength and endurance. Differences between the groups in these two measures were statistically significant, and poorer scores were achieved by the overweight and obese adolescents. The differences in flexibility and aerobic capacity between the children with normal weight and those with overweight or obesity were not statistically significant. The relationship between obesity and physical fitness in adolescents was also assessed by Salaun et al. In that study, the only significant difference was identified in the measure of trunk muscle strength between girls of normal weight and girls with obesity^19^.

While discussing these findings, it should be emphasised that this subject matter re-quires a great deal of attention as it is important for designing preventive measures and rehabilitation programmes tailored to the needs of children with ID, taking into account the BMI percentile. The findings showed that all children with mild ID, whether presenting with obesity or normal weight, should have an opportunity to participate in adequate rehabilitation programmes, as these lead to improvements in physical fitness and static balance, a fact that should be considered in all special education centres.

## Limitations

This study presents some limitations. Firstly, it did not take into account a no-treatment control group of healthy children or children with mild ID, which would have made it possible to control more variables and attribute the changes to the intervention. Secondly, the findings are only applicable to children with mild ID. Hence, further research should assess the response of children with moderate and severe ID to the rehabilitation programme. Furthermore, this study involved a narrow age cohort of children with mild ID (aged 11–15 years). Hence, in the next stage of research, other age groups should also be assessed for the effects of the therapy programme. It will also be necessary to add more exercises that enable improvements in dynamic balance and muscular endurance into the rehabilitation programme, and to increase the intensity and frequency of exercise. Balance is important for children with ID, not only as a skill component, but also for health-related fitness. The present study focused exclusively on assessing static/dynamic balance reflected by specific physical skills; therefore, further research should be designed to also assess balance as a factor impacting health-related fitness. Moreover, future studies should utilise new research tools and questionnaires assessing physical fitness in relation to independence, as well as functioning in daily life. Furthermore, in future research, the rehabilitation programme should be redesigned to include incentivising and motivating components, and to match the capacities of children with ID. Ultimately, a follow-up study that includes a comparative analysis of different ethnicities from various continents could be conducted to broaden the subject matter of the research in the future.

## Conclusions

This study showed that a six-month rehabilitation programme positively impacted the lower and upper limb muscle strength, flexibility, and static balance of an entire cohort of children with mild ID. The effects of rehabilitation on the measures of static and dynamic balance and physical fitness did not differ significantly between the children with obesity and those with normal weight. The findings suggest that all children with mild ID, whether presenting with obesity or normal weight, can achieve improvements in physical fitness and static balance if they have an opportunity to participate in this type of rehabilitation programme. This evidence is important from the viewpoint of clinical practice and, even more so, for preventive measures implemented in special education centres. Therefore, this information should be taken into account by those designing therapeutic programmes intended for children with mild ID that are provided in special education centres. Further research related to this subject matter should focus on children with moderate to severe ID, more age groups, and the long-term effects of the programme.

## Data Availability

The datasets used and/or analysed during the current study are available from the corresponding author on reasonable request.

## References

[1] Fredericks DW, Williams WL (1998). New definition of mental retardation for the American association of mental retardation. Image J. Nurs. Sch..

[2] Wieczorek M (2008). Physical fitness in mentally disabled youth as the factor conditioning their health. Probl. Hig. Epidemiol..

[3] Patel DR, Apple R, Kanungo S, Akkal A (2018). Intellectual disability: definitions, evaluation and principles of treatment. Pediatr. Med..

[4] Wolan-Nieroda A, Dudziak J, Drużbicki M, Pniak B, Guzik A (2021). Effect of dog-assisted therapy on psychomotor development of children with intellectual disability. Children.

[5] Xu C, Yao M, Kang M, Duan G (2020). Improving physical fitness of children with intellectual and developmental disabilities through an adapted rhythmic gymnastics program in China. Biomed. Res. Int..

[6] Valentin-Gudiol M (2011). Treadmill interventions with partial body weight support in children under six years of age at risk of neuromotor delay. Cochrane Database Syst. Rev..

[7] Boer PH (2014). The influence of sprint interval training on body composition, physical and metabolic fitness in adolescents and young adults with intellectual disability: A randomized controlled trial. Clin. Rehabil..

[8] Elmahgoub SM, Lambers S, Van Laethem SSCh, Cambier D, Calders P (2009). The influence of combined exercise training on indices of obesity, physical fitness and lipid profile in overweight and obese adolescents with mental retardation. Eur. J. Pediatr..

[9] Wu WL (2017). Effectiveness of a cross-circuit exercise training program in improving the fitness of overweight or obese adolescents with intellectual disability enrolled in special education schools. Res. Dev. Disabil..

[10] Gupta S, Rao BK (2011). Effect of strength and balance training in children with Down's syndrome: A randomized controlled trial. Clin. Rehabil..

[11] Golubović S, Maksimović J, Golubović B, Clumbić N (2012). Effects of exercise on physical fitness in children with intellectual disability. Res. Dev. Disabil..

[12] Frey GC, Chow B (2006). Relationship between BMI, physical fitness, and motor skills in youth with mild intellectual disabilities. Int. J. Obes. Lond..

[13] Gawlik K, Zwierzchowska A, Rosołek B, Celebańska D, Franusz G (2020). Excessive body weight versus physical fitness in adults with moderate and severe intellectual disabilities. Szk. Spec..

[14] Giagazoglou P (2013). Effects of a trampoline exercise intervention on motor performance and balance ability of children with intellectual disabilities. Res. Dev. Disabil..

[15] Giagazoglou P, Arabatzi F, Dipla K, Liga M, Kellis E (2012). Effect of a hippotherapy intervention program on static balance and rhythmic gymnastics^12^ength in adolescents with intellectual disabilities. Res. Dev. Disabil..

[16] Chao-Chien Ch, Yi-Chun L (2012). Jumping rope intervention on health-related physical fitness in students with intellectual impairment. J. Hum. Resour. Manag..

[17] Gąsior J, Pawłowski M, Bonikowski M, Jeleń P, Błaszczyk J (2013). Strength training as a form of rehabilitation in children and adolescents with cerebral palsy: A review. Child Neurol..

[18] Golubović S, Maksimović J, Golubović B, Clumbić N (2012). Effects of exercise on physical fitness in children with intellectual disability. Res. Dev. Disabil..

[19] Salaun L, Berthouze-Aranda SE (2012). Physical fitness and fatness in adolescentswith intellectual disabilities. J. Appl. Res. Intellect. Disabil..

[20] Flanagan, D. P. & Kaufman, A.S. *Essentials of WISC-IV Assessment*. Hoboken. (ed. Flanagan, D. P., Kaufman, A.S.) 31–41 (John Wiley and Sons, NJ, 2004).

[21] Kalkulator OLAF. Available online: http://olaf.czd.pl/index.php?option=com_content&view=article&id=103:kalkulator (2022).

[22] Centers for Disease Control and Prevention. Available online: https://www.cdc.gov/healthyweight/assessing/bmi/childrens_bmi/about_childrens_bmi.html (2022).

[23] Skowroński W (2007). Eurofit Specjalny Motor Fitness Test for People with Intellectual Disability.

[24] Drobnik P, Cybulska A, Dargiewicz R (2015). The author’s programme of motoral improvement and physical fitness of intellectually disabled people. Physiotherapy.

[25] Condon C, Cremin K (2014). Static balance norms in children. Physiother. Res. Int..

[26] Itzkowitz A (2016). Timed up and go: Reference data for children who are school age. Pediatr. Phys. Ther..

[27] Kachouri H (2016). The effect of a combined strenght and proprioceptive training on muscle strength and postural balance in boys with intellectual disability: An exploratory study. Res. Dev. Disabil..

[28] Pejčić A, Kocić M, Berić D (2019). The effects of special sports games program on physical fitness in adolescents with intellectual disability. Acta Fac. Med. Naissensis..

[29] Van De Vliet P (2006). Physical fitness profile of elite athletes with intellectual disability. Scand. J. Med. Sci. Sports..

[30] Dehghani M, Gunay M (2015). The effect of balance training on static and dynamic balance in children with intellectual disability. J. Appl. Environ. Biol. Sci..

[31] Zolghadr H, Sedaghati P, Daneshmandi H (2019). The effect of eight weeks of selected balance-corrective exercises on motor performance of students with mental retardation with developmental coordination disorder. Phys. Treat..

[32] Jeng SC (2013). A physical fitness follow-up in children with cerebral palsy receiving 12-week individualized exercise training. Res. Dev. Disabil..

[33] Muehlbauer T (2021). Effects of balance training on static and dynamic balance performance in healthy children: Role of training duration and volume. BMC Res. Notes..

[34] Schedler S, Brock K, Fleischhauer F, Kiss R, Muehlbauer T (2020). Effects of balance training on balance performance in youth: Are there age differences?. Res. Q Exerc. Sport..

[35] Lengkana AS (2000). Static and dynamic balance learning in primary school students. Int. J. Hum. Mov. Sports Sci..

[36] Matsunaga N (2022). Decreased balance function in school-aged children with behavioral problems. Brain Sci..

[37] Bozkurt S, Erkut O, Akkoç O (2017). Relationships between static and dynamic balance and anticipation time, reaction time in school children at the age of 10–12 years. Univ. J. Educ. Res..

[38] Mikołajczyk E, Jankowicz-Szymańska A (2017). Dual-task functional exercises as an effective way to improve dynamic balance in persons with intellectual disability—Continuation of the project. Med. Stud..

[39] Singh B (2021). The effects of adiposity, muscular strength, cardiorespiratory fitness, and fatigue on gait biomechanics in overweight and obese children. Clin. Biomech. Bristol. Avon..

[40] Gushue D, Houck J, Lerner A (2005). Share effects of childhood obesity on three-dimensional knee joint biomechanics during walking. J. Pediatr. Orthop..

[41] Lerner ZF, Shultz SP, Board WJ, Kung S, Browning RC (2014). Does adiposity affect muscle function during walking in children?. J. Biomech..

